# Divergent pandemic responses in miscarriage and ectopic pregnancy admissions

**DOI:** 10.1016/j.lanepe.2026.101875

**Published:** 2026-09-22

**Authors:** Neil Cockburn, Joht Singh Chandan, William Parry-Smith

**Affiliations:** a Warwick Applied Health, University of Warwick, Coventry, UK; b Nuffield Department of Primary Care Health Sciences, University of Oxford, Oxford, UK; c Department of Obstetrics and Gynaecology, Shrewsbury and Telford Hospitals NHS Trust, Telford, UK; d School of Medicine, Keele University, Keele, UK

Sekar and colleagues report 20-year time series in miscarriage and ectopic pregnancy admissions in England, and their contribution highlights important trends that they rightly emphasise are not routinely monitored.^1^ The two conditions also differ in what drives admission, with revealing differences in pandemic responses.

Miscarriage admissions fell 14% in a single pandemic year, remained low through 2022 and 2023, and in 2024 returned to within 1% of their 2019 level, still below every year from 2005 to 2018. Conceptions meanwhile rose in 2021, 2022 and 2023.^2^ A single year recovering to the pre-pandemic level is more consistent with restored care-seeking and admission thresholds than with a change in underlying risk.

Ectopic admissions were unmoved by the pandemic, exceeded their pre-pandemic level between 2022 and 2024, and reached a 20-year maximum around 20% above the 2012–2020 trajectory, while deliveries fell.^3^ The long-run rise, corroborated by primary care data showing incidence in England increasing from 38.0 to 44.1 per 100,000 person-years between 2006 and 2021,^4^ appears to be accelerating. Improvements in diagnostic sensitivity accrue gradually and would not produce a decade of stability followed by a step change.

The contrast suggests concerning change in ectopic admissions, and for caution in reading miscarriage admissions as rising incidence.

## Contributors

All authors contributed equally.

## Declaration of generative AI and AI-assisted technologies in the manuscript preparation process

During the preparation of this work, the authors used Claude Opus 5.0 for proof reading and text searching against the original article. The authors reviewed and edited the output as needed and take full responsibility for the content of the published article.

## Declaration of interests

NC and colleagues have a related manuscript on the incidence of ectopic pregnancy in England under consideration at another journal. We otherwise declare no competing interests.
